# Plant Growth-Promoting Methylobacteria Selectively Increase the Biomass of Biotechnologically Relevant Microalgae

**DOI:** 10.3389/fmicb.2020.00427

**Published:** 2020-03-18

**Authors:** Lisa Krug, Christina Morauf, Christina Donat, Henry Müller, Tomislav Cernava, Gabriele Berg

**Affiliations:** ^1^Institute of Environmental Biotechnology, Graz University of Technology, Graz, Austria; ^2^acib GmbH, Graz, Austria; ^3^bio-ferm GmbH, Getzersdorf, Austria

**Keywords:** microalgae, microbiota, growth-promotion, methylobacteria, symbiosis, algae–bacteria interactions

## Abstract

Microalgae, a diverse group of single-celled organisms exhibiting versatile traits, find broad applications in industry. However, high production costs require further efforts to optimize their production and to enhance biomass yields. In the present study, co-occurrence of algae and methylobacteria was observed when naturally occurring microalgae biofilms were subjected to 16S rRNA gene fragment amplicon sequencing. This bacterial group is so far less explored than other microalgae-associated bacteria in terms of mutualistic relationships that might be exploitable for biotechnological applications. In order to assess the potential of four plant growth-promoting strains from the genus *Methylobacterium* for increased algae biomass production, co-cultivation experiments were conducted with three industrially relevant microalgae (*Chlorella vulgaris*, *Scenedesmus vacuolatus*, and *Haematococcus lacustris)*. For *S. vacuolatus* and *H. lacustris*, a significant increase in algal biomass formation of 1.3-fold to up to 14-fold was observed after 7 days of co-incubation. Visualization of mixed cultures using confocal laser scanning microscopy revealed a high abundance of methylobacteria in the phycosphere of *H. lacustris* and *S. vacuolatus*, visually attached to the algae’s surface forming a biofilm-like assemblage. Genome analyses revealed that features attributable to enhanced algal growth include genes involved in the synthesis of vitamins, siderophores and plant hormones. Our results provide evidence for the constructability of novel symbiotic algae-bacteria relationships with inter-kingdom supportive capacities, underlining the potential of microbial consortia as promising tool for sustainable biotechnology and agriculture.

## Introduction

In the recent past, the potential of microalgae for industrial purposes increasingly gained importance at a global scale. Due to their versatile characteristics including high lipid content and their ability to accumulate high-value compounds, they can be used as supplements for food and animal feed, as pharmaceuticals, as feedstock for biofuel production, or as sustainable alternative to synthetic fertilizers (Spolaore et al., 2006; Brennan and Owende, 2010; Renuka et al., 2018). In order to meet industrial demands, microalgae are cultivated either in open pond systems for bulk material production or in closed photobioreactors for the production of high-value compounds. However, both cultivation methods often fail to reach economic competitiveness in terms of biomass yields, contamination prevention and the involved costs (Benemann and Oswald, 1996; Wang et al., 2013). Therefore, further efforts need to be made in order to reduce costs and enhance biomass yields by optimizing cultivation conditions for efficient microalgae production that can compete with non-sustainable alternatives. One means of increasing microalgae populations and thus enhancing yields might be the co-inoculation with beneficial microorganisms as already successfully applied in agriculture for increasing yields of vascular land plants (Berg, 2009; Lugtenberg and Kamilova, 2009; Berg et al., 2013). This strategy is primarily applicable for large-scale production where cultivation conditions are not optimal and contaminations with other microorganism are unavoidable. Algae were shown to benefit from micro- and macronutrients provided by bacteria, and in return, release dissolved organic carbon into their surroundings. Efforts have been made in identifying bacteria that positively contribute to the algal fitness; especially nitrogen-fixing bacteria of the genera *Azospirillum, Rhizobium*, and *Bacillus*, known for their beneficial effect on plants, are promising candidates (Gonzalez and Bashan, 2000; de-Bashan et al., 2008; Hernandez et al., 2009; de-Bashan and Bashan, 2010; Kim et al., 2014; Amavizca et al., 2017). While binary algae-bacteria interactions were explored in the past, little is known about the algae-associated microbiota as well as potentially beneficial algae-microbe interactions with distinct members of the plant microbiota.

The species-specific plant microbiota forms complex networks and contributes multiple aspects to the functioning of the plant holobiont, such as (i) seed germination and growth, (ii) nutrient supply, (iii) resistance against biotic and abiotic stress factors, and (iv) production of bioactive metabolites (Berg et al., 2017). Mutualistic inter-kingdom symbioses are not only limited to land plants, but also extend to green algae (Xie et al., 2013; Hom et al., 2015). Following the same principles of plant growth-promoting rhizobacteria, algae-associated bacteria harbor potential to stimulate growth and morphogenesis of algae by releasing essential minerals, vitamins, auxins and quorum sensing signaling molecules (Joint et al., 2002; Croft et al., 2006; Goecke et al., 2010; Amavizca et al., 2017). The equivalent to the rhizosphere – the immediate surrounding of roots with increased biological and chemical activity in soil – is the phycoscphere, which is the area surrounding a phytoplankton cell, rich in dissolved organic matter and metabolites exuded by the cell in the surrounding water and stimulating the growth of heterotrophic bacteria (Seymour et al., 2017). Compared to the plant rhizosphere, less is known about the structure and function of the phycosphere microbiota, although studies show negative (Hom et al., 2015) and positive impacts of bacteria on algal growth (Croft et al., 2005; Amin et al., 2009; Hernandez et al., 2009; Kim et al., 2014; Cooper and Smith, 2015).

In order to deepen our understanding of the phycosphere microbiota, we analyzed the naturally occurring microbiome of algae in order to detect potential algae-bacteria associations. Based on the preliminary findings we decided to focus on a bacterial group known for its symbiotic interactions with agricultural plants and three eukaryotic model organisms representing industrially relevant microalgae. The microalgae *Chlorella vulgaris*, *Scenedesmus vacuolatus* and *Haematococcus lacustris* are used as feedstock for biofuel production due to their high amount of carbohydrates and as source material for the production of astaxanthin, a strong antioxidant used in food and pharmaceutical industry (Sharma and Sharma, 2017; Khan et al., 2018). In order to explore the transferability of beneficial, host-associated microorganisms, methylobacteria that are already known for their plant-beneficial traits, were selected from our in-house strain collection. The selected isolates belong to three different species, *Methylobacterium extorquens, M. mesophilicum*, and *M. goesingense*, and were originally isolated from different plant species (Verginer et al., 2010). By implementing co-cultivation experiments, the potential of methylobacteria to induce and promote the growth of microalgae was evaluated. In order to provide deeper insights into the mechanisms and bacterial features associated with growth-promoting effects, the whole genomes of *M. extorquens* Rab1 and *M. mesophilicum* Sab1 were sequenced and analyzed. The obtained results provide the background for novel co-cultivation approaches that make use of inter-kingdom supporting capacities of beneficial, plant-associated bacteria.

## Materials and Methods

### Sampling Procedure

Due to the generally higher prevalence beneficial interactions in nutrient-poor systems, naturally occurring algae communities from artificial surfaces were selected for phycobiome analyses. Samples were collected on November 1st 2016 by removing a natural biofilm on the surface of outdoor furniture in Graz (Austria; 47° 4′′ N, 15° 26′′ O) at an altitude of 353 m above sea level, in three replicates, where macroscopic observations and coloration of biofilm indicated the occurrence of microalgae (Supplementary Figure S1A). Upon arrival in the laboratory, samples were resuspended in 3 mL sterile NaCl (0.85%) and visualized microscopically using a light microscope (Leitz, Wetzlar, Germany) at 200 × magnification in order to confirm the occurrence of microalgae (Supplementary Figure S1B).

### Isolation and Identification of Microalgae

In order to isolate microalgae, the aforementioned samples were plated in dilution series on modified Bolds Basal Medium agar plates (BBM) containing 250 mg/L NaNO_3_, 175 mg/L KH_2_PO_4_, 75 mg/L K_2_HPO_4_, 75 mg/L MgSO_4_ × 7 H_2_O, 25 mg/L CaCl_2_, 25 mg/L NaCl, 2.6 mg/L H_3_BO_3_, 5 mg/L FeSO_4_ × 7 H_2_O, 8.8 mg/L ZnSO_4_ × 7 H_2_O, 1.4 mg/L MnCl_2_ × 4 H_2_O, 1.4 mg/L MoO_3_, 1.6 mg/L CuSO_4_ × 5 H_2_O, 0.5 mg/L Co(NO_3_)_3_ × 6 H_2_O, 0.5 mg/L EDTA, 0.3 mg/L KOH, 0.017 mg/L vitamin B_12_, 0.013 mg/L 4-aminobenzoate, 0.003 mg/L biotin, 0.013 mg/L nicotinic acid, 0.017 mg/L hemicalcium-pentathenate, 0.05 mg/L pyridoxamine-HCl, 0.033 mg/L thiaminiumdichlorid, 0.0091 mg/L thioctic-acid, 0.01 mg/L riboflavin, 0.0049 mg/L folic acid and 18 g/L agar-agar. Vitamins and heat sensitive components were added after autoclaving by sterile filtration (0.20 μm pore size). In order to obtain pure cultures single algae colonies were repeatedly subcultured on modified BBM-agar and incubated at 23°C at a light dark cycle (L:16/D:8). The lighting was supplied by cool-white fluorescent lamps TL-D 36W/840 REFLEX Eco (Philips, Amsterdam, Netherlands) with a photosynthetic photon flux of 115 μmol s^–1^.

In order to identify isolated microalgae species, cells were resuspended in 300 μL NaCl (0,85%) and transferred in sterile Eppendorf tubes filled with glass beads. After mechanical disruption using a FastPrep FP120 instrument (MP Biomedicals, Germany) suspensions were centrifuged at 3,000 rpm for 5 min. Supernatant served as template for the following PCR reaction. Partial 18S rRNA gene fragments were amplified using primer pair NS1 (5′-GTA GTC ARA RGC CTT GTC TC-3′) and NS8 (5′-TCC GCA GGT TCA CCT ACG GA-3′) in a reaction mix (30 μL) containing 16.2 μL ultrapure H_2_O, 6 μL Taq&Go [5×], 1.2 μL of each primer [10 μM], 2.4 μL MgCl_2_ [25 mM] and 3 μL DNA template. The cycling program was adjusted to the following settings: 95°C, 10 min; 40 PCR products were then purified using the Wizard SV Gel and PCR-Clean-Up System (Promega Corporation, Madison, WI, United States) according to manufacturer’s protocol. The amplified 18S rRNA gene fragments were sequenced by Sanger sequencing (LGC genomics; Berlin, Germany) and subsequently aligned against the NCBI nucleotide collection database for taxonomic assignments. Microscopic visualization of the pure cultures was used to verify the absence of other microorganisms in the pure cultures of the isolated microalgae *C. vulgaris* G1-G, *S. vacuolatus* G1-O and *H. lacustris* G1-R (Supplementary Figure S2).

### Algae-Associated Microbiome Analyses Using 16S rRNA Gene Fragment Amplicon Libraries

#### Total Community DNA Extraction and Barcoding

Total community DNA of three biological replicates was extracted using the FastDNA Kit for Soil (MP Biomedicals, Heidelberg, Germany) according to the manufacturer’s protocol. The 16S rRNA gene fragments were amplified in three technical replicates covering the hypervariable region 4 using the Unibac II 515f (5′-GTG YCA GCM GCC GCG GTA A-3′) and 806r (5′-GGA CTA CHV GGG TWT CTA AT-3′) primer pair (Caporaso et al., 2011), which included sample-specific barcodes and Illumina sequencing adaptors. Peptide nucleic acid (PNA) was added to the PCR mix to prevent the amplification of mitochondrial (mPNA) and plastidial (pPNA) DNA from eukaryotes (Lundberg et al., 2013). The PCR was performed by using a total volume of 30 μL containing 20.15 μL ultrapure water, 6 μL Taq&Go [5×], 1.2 μL of each primer (5 μM), 0.225 μL pPNA [100 μM], 0.225 μL mPNA [100 μM] and 1 μL DNA template. The cycling program was adjusted to an initial denaturation temperature at 96°C for 5 min, followed by 30 cycles of 96°C for 1 min, 78°C for 5 s, 54°C for 1 min, and 74°C for 1 min. The final extension was done at 74°C for 10 min. The PCR products of all samples were quality checked by gel electrophoresis. Subsequently, the PCR products were purified using the Wizard SV Gel and PCR-Clean-Up System according to manufacturer’s protocol. Equimolar DNA concentrations of each barcoded amplicon were sent for paired end Illumina HiSeq sequencing (read length: 2 × 300 bp) to GATC Biotech AG (Konstanz, Germany).

#### Bioinformatic Analyses of 16S rRNA Gene Fragments Amplicons

Initial data processing, including joining of forward and reverse read pairs was done using software package QIIME 1.9.1 (Caporaso et al., 2010). After removing barcodes, primer and adapter sequences reads as well as metadata were imported into QIIME 2 (2018.11 release). Further analyses of sequencing data were performed using the QIIME 2 pipeline according to tutorials provided by the QIIME developers (Caporaso et al., 2010). The DADA2 algorithm (Callahan et al., 2016) was used to demultiplex and denoise truncated reads and remove chimeras. Taxonomic analyses are based on a Naïve-Bayes classifier trained on the SILVA 128 release database (Quast et al., 2013) clustering at 99% similarity. After removing mitochondrial, chimeric and plastid sequences, the 16S rRNA dataset was normalized to 264,542 reads. A normalized feature table served as input for the OTU table (make_out_network.py) using QIIME 1.9.1. OTU-network was generated and rendered using Cytoscape version 3.7.0 (Shannon et al., 2003). Features assigned to the genus *Methylobacterium* were manually aligned against the NCBI nucleotide collection using the BLAST algorithm (Altschul et al., 1990).

### Bacterial Strains and Initial Culture Conditions

For growth assays *M. extorquens* Rab1 (deposited at DSMZ; DSM 21961), *M. mesophilicum* Sab1 (deposited at DSMZ; DSM 21962), *M. goesingense* Vab1 and *M. goesingense* Vab2 were selected from the in-house strain collection SCAM (Strain Collection of Antagonistic Microorganisms; Institute of Environmental Biotechnology, Graz University of Technology). They were originally isolated from different plant species (Verginer et al., 2010). Bacteria were cultured on MIS (methanol, inorganic salt) agar plates containing 1.8 g/L (NH_4_)_2_SO_4_, 0.2 g/L MgSO_4_ × 7 H_2_O, 1.4 g/L NaH_2_PO_4_ × 2 H_2_O, 1.9 g/L K_2_HPO_4_ and 18 g/L agar-agar. After autoclaving 5 mL/L methanol and 1 mL/L sterile filtered (0.20 μm pore size) trace element solution containing 500.0 mg/L EDTA, 200.0 mg/L FeSO_4_ × 7 H_2_O, 10.0 mg/L ZnSO_4_ × 7 H_2_O, 3.0 mg/L MnCl_2_ × 4 H_2_O, 30.0 mg/L H_3_BO_3_, 20.0 mg/L CoCl_2_ × 6H_2_O, 1.0 mg/L CuCl_2_ × 6 H_2_O, 2.0 mg/L NiCl_2_ × 6 H_2_O, and 3.0 mg/L Na_2_MoO_4_ × 2 H_2_O were added.

### Evaluation of Microalgae Growth-Promoting Effects of *Methylobacterium* spp. Through Co-cultivation Experiments

The effect of four different methylobacteria strains on the growth of three different microalgal genera, including *Chlorella, Scenedesmus* and *Haematococcus* was evaluated through co-cultivation experiments. In order to explore the effect of the bacterial load on the performance of the microalgae, bacteria were added in two different cell concentrations (OD_600_ 0.2 and OD_600_ 0.5) at the beginning of the experiments. For the preparation of bacterial pre-cultures, 100 μL of the respective bacterial suspension were plated on 15–20 MIS agar plates. After 7 days of incubation at 30°C bacterial lawn was harvested using sterile object slide, resuspended in NaCl (0.85%) and used to reach the targeted absorbance OD_600_ of 0.5 and 0.2 in the main cultures. For *C. vulgaris* G1-G and *S. vacuolatus* G1-O, co-culture experiments were performed in 12 replicates in Parafilm^®^ -sealed 12-well plates containing a final volume of 3 mL vitamin-depleted BBM medium (dBBM) containing 250 mg/L NaNO_3_, 175 mg/L KH_2_PO_4_, 75 mg/L K_2_HPO_4_, 75 mg/L MgSO_4_ × 7 H_2_O, 25 mg/L CaCl_2_, 25 mg/L NaCl, 2.6 mg/L H_3_BO_3_, 5 mg/L FeSO_4_ × 7 H_2_O, 8.8 mg/L ZnSO_4_ × 7 H_2_O, 1.4 mg/L MnCl_2_ × 4 H_2_O, 1.4 mg/L MoO_3_, 1.6 mg/L CuSO_4_ × 5 H_2_O, 0.5 mg/L Co(NO_3_)_3_ × 6 H_2_O, 0.5 mg/L EDTA, 0.3 mg/L KOH. Algal pre-cultures were obtained by inoculating 50 mL BBM with a single colony of the respective microalgae. After a sufficient cell density was reached, these pre-cultures served as inoculum for co-cultivation experiments to reach a FI of 154 ± 29, which corresponds to 5.08 ± 0.94 × 10^4^ CFU/mL for *C. vulgaris* G1-G and to a FI of 152 ± 21, corresponding to 3.51 ± 0.47 × 10^4^ CFU/mL for *S. vacuolatus* G1-O. For *H. lacustris* G1-R, the co-culture experiments were performed in 12 replicates in sterile 100-mL flasks sealed with aluminum foil containing a total volume of 10 mL dBBM. Algal pre-cultures were obtained by rinsing two BBM agar plates containing *H. lacustris* cultures with dBBM. The FI was adjusted to 33 ± 8 at the beginning of the experiments, corresponding to 7.04 ± 1.65 × 10^3^ algal CFU/mL.

All co-cultures were incubated at 23°C at a light dark cycle (L:16/D:8). The lighting was supplied by cool-white fluorescent lamps TL-D 36W/840 REFLEX Eco (Philips, Amsterdam, Netherlands) with a photosynthetic photon flux of 115 μmol s^–1^. Biomass formation was determined by measuring the fluorescence intensity at 685 nm when excited at 450 nm using an infinite M200 spectrofluorimeter (TECAN; Switzerland) after 7 days for all included algae and additionally after 14 days for *C. vulgaris* and *S. vacuolatus*. The second measurement (14 days) was not conducted for *H. lacustris* G1-R due to the onset of encapsulation. In order to correlate the FI with algal cell counts, fluorescence measurements and subsequent plating of the respective dilutions on BBM agar were conducted. Linear regression allowed the correlation between FI and microalgae cell counts (*C. vulgaris* G1-G: *R*^2^ = 0.95, Supplementary Figure S3A; *S. vacuolatus* G1-O: *R*^2^ = 0.98, Supplementary Figure S3B; *H. lacustris* G1-R: *R*^2^ = 0.87; Supplementary Figure S3C).

### Visualization of Co-cultures

All microscopic visualizations were done using a Leica TCS SPE confocal laser scanning microscope (Leica Microsystems GmbH, Mannheim, Germany) and the oil immersion objectives Leica ACS APO 40.0 × 1.15 (183.33 μm × 183.33 μm) and ACS APO 63 × 1.30 (116.40 μm × 116.40 μm). Solid-state lasers were used with 405, 488, 532, 635 nm excitation. The micrograph included in the OTU network was obtained with the natural community within the sampled biofilm following staining with the LIVE/DEAD bacterial viability kit (Thermo Fisher Scientific, MA, United States) in combination with calcofluor white (0.15%; Sigma-Aldrich, Missouri, United States). Micrographs of co-cultures were obtained with *M. goesingense* Vab1 and *C. vulgaris*, *S. vacuolatus* and *H. lacustris*, respectively, with an initial bacterial optical density of 0.5 after 7 days of incubation. For visualization of mixed cultures, 1 mL of the respective cultures were transferred into 1.5 mL reaction tubes and let stand for 2–3 h to allow sedimentation of the microorganisms. The supernatant was discarded and the remaining cultures were stained using the LIVE/DEAD bacterial viability kit in combination with calcofluor white at a concentration of 0.15%.

### Genomic DNA Extraction

*Methylobacterium extorquens* Rab1 and *M. mesophilicum* Sab1 were grown on solid MIS medium and incubated for 7 days at 30°C. Cells were then collected from the plates using a sterile pipet tip and resuspended in 500 μL sterile NaCl (0.85%). Genomic DNA was extracted using the MasterPure DNA purification kit (Epicentre, WI, United States). DNA quality and quantity were checked by agarose gel electrophoresis, fluorometry (Qubit 4, Thermo Fisher Scientific, MA, United States) and spectrophotometry using a UV-Vis spectrophotometer (NanoDrop 2000c, Thermo Fisher Scientific, MA, United States). Subsequently, genomic DNA of *M. extorquens* Rab1 (5.05 μg; 0.19 μg/mL) and *M. mesophilicum* Sab1 (8.02 μg; 0.30 μg/mL) was sent for Illumina NextSeq 500/550 V2 (150 bp paired-end sequencing; LGC Genomics GmbH, Berlin, Germany).

### Genome Assembly and Annotation

The genomes of *M. extorquens* Rab1 and *M. mesophilicum* Sab1 were assembled using SPAdes (Bankevich et al., 2012) and arranged into scaffolds. The scaffolds were then reassembled by using reference genomes (*M. extorquens* Rab1: NZ_CP021054.1; *M. mesophilicum* Sab1: NC_010505.1) and AlignGraph (Bao et al., 2014) in order to extend and join scaffolds. The annotation was done by using the Rapid Annotation using Subsystem Technology (RAST; Aziz et al., 2008).

### Statistical Analyses

Each co-cultivation experiment was conducted with 12 replicates that were separately assessed. The resulting data was tested for normal distribution in order to select appropriate statistical tests. Significances were then determined using ANOVA for normally distributed values and the Kruskal-Wallis test for non-parametric analyses including Bonferroni multiple test correction.

## Results

### Microalgae and Methylobacteria Naturally Co-occur in Mixed-Community Biofilms

**FIGURE 1:**
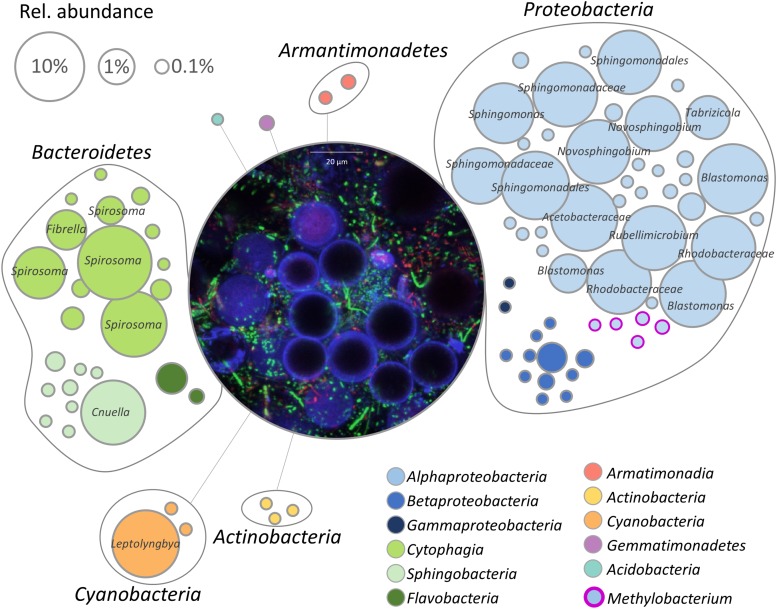
Network visualization of the bacterial community within the biofilm. Each node represents a feature (99% cut-off level), whereas the node size corresponds to their relative abundance. Taxonomy of highly abundant features (>1%) is included. The central micrograph shows the natural microbial community within the biofilm after staining with the LIVE/DEAD bacterial viability kit in combination with calcofluor white (0.15%). Blue: algal cell wall (cellulose, chitin), green: viable bacterial cells, red: dead bacteria.

The bacterial fraction occurring in the microalgae-dominated biofilm was investigated by 16S rRNA gene fragment amplicon sequencing. After removal of chimeric, mitochondrial and chloroplast sequences, 264,542 reads remained in the dataset, resulting in 92 features. The bacterial microbiome consisted of seven phyla with *Proteobacteria* (61%) being the most dominant followed by *Bacteroidetes* (32%) and *Cyanobacteria* (7%). *Acidobacteria*, *Actinobacteria*, *Armatimonadetes*, and *Gemmatimonadetes* occurred in relative abundance less than 1%. On class level, *Alphaproteobacteria* (58%) were the dominating group followed by *Cytophagia* (27%) and *Cyanobacteria* (7%); *Sphingobacteriia* (4%), *Betaproteobacteria* (2%), *Flavobacteria* (1%), *Acidobacteria* (<1%), *Actinobacteria* (<1%), *Armatimonadia* (<1%), and *Gammaproteobacteria* (<1%) occurred in a substantially lower relative abundance. Members of the order *Sphingomonadales* (41%) were most frequently detected, followed by *Cytophagales* (27%), *Rhodobacteriales* (9%), and *Rhodospirillales* (8%). In total, 49 bacterial genera were identified with *Spirosoma* (24%) being the dominating fraction, followed by *Porphyrobacter* (11%) and not further classified *Sphingomonadales* (11%). Despite *Methylobacterium* spp. accounted only for a low proportion of the total bacterial community, their occurence in the core microbiome indicates that they are potentially embedded in a mutualistic interaction with microalgae (Figure 1). Five distinct features in the analyzed sample represented the genus and were also manually aligned against the NCBI nt collection; Supplementary Table S1 lists respective feature ID and closest hit after BLAST search. Visualization of the natural biofilm using a confocal laser scanning microscope revealed *H. lacustris* as the dominating algal taxon (Figure 1 and Supplementary Figure S1).

### Co-cultivation of Distinct Microalgae and Methylobacteria Leads to Significantly Increased Biomass Formation

**FIGURE 2:**
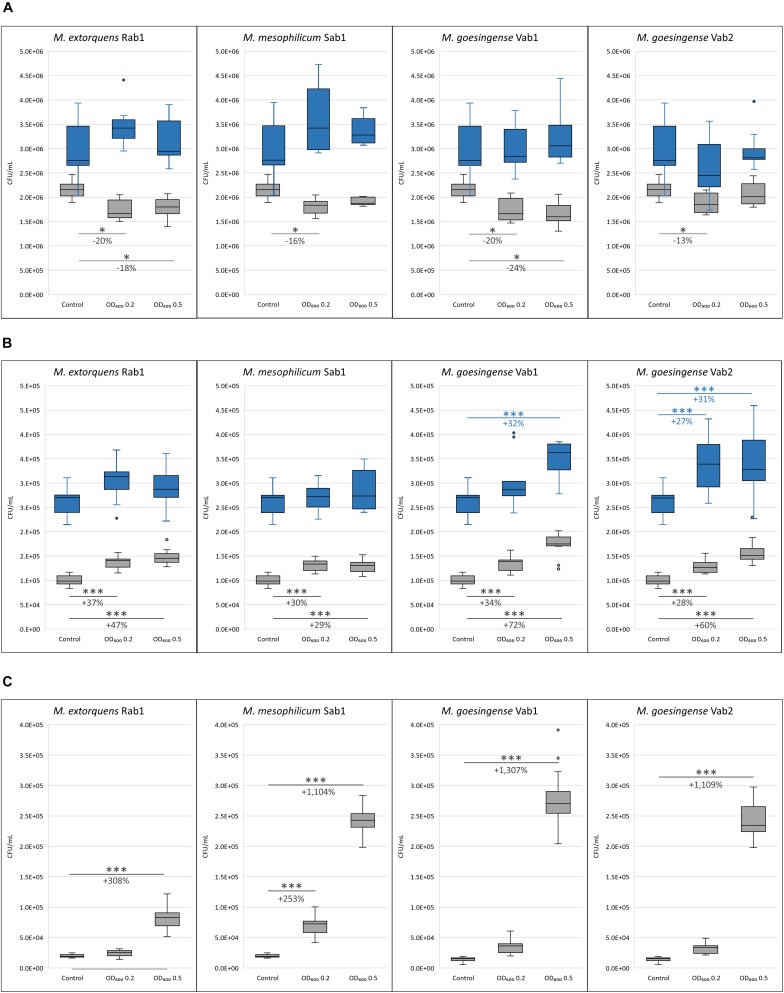
Microalgal cell counts after seven (grsy boxes) and 14 (blue boxes) days of incubation. *C. vulgaris* G1-G **(A)**, *S. vacuolatus* G1-O **(B)**, and H. *lacustris* G1-R **(C)** were co-inoculated with four different *Methylobacterium* strains in two differing cell concentrations (initial cell densities: OD_600_ 0.2 and 0.5). Algal CFU was determined by measuring the fluorescence intensity (FI). For each box, the central line indicates the median, while the bottom and top edges of the box indicate the 25th and 75th percentiles, respectively. The whiskers extend to the most extreme data points not considered outliers. Asterisks indicate significant differences in algal cell count compared to the control (^∗^*p*-value ≤ 0.05; ^∗∗^*p*-value ≤ 0.01; ^∗∗∗^*p*-value ≤ 0.001). The percentage of increased/reduced microalgal cell counts compared to the control is included.

Co-cultivation experiments revealed varying effects of methylobacteria on the growth of different microalgal genera. While co-cultivation with methylobacteria led to a significant decrease in algal cell count for *C. vulgaris* G1-G, contrary effects were revealed for *S. vacuolatus* G1-O and *H. lacustris* G1-R. Both, growth-promoting as well as growth-inhibiting effects of methylobacteria were stronger after 7 days of co-cultivation but decreased after 14 days of incubation. In general, the growth-promotion by methylobacteria was more explicit, when the bacterial population density was higher at the beginning of the experiments. In detail, a significant decrease in microalgal biomass was observed for *C. vulgaris* G1-G when co-cultured with all tested methylobacteria with an initial optical density of 0.2 after 7 days of incubation compared to the axenic control. For *M. extorquens* Rab1 and *M. goesingense* Vab1 a significant decrease in microalgal biomass formation was also observed when the initial bacterial cell density was adjusted to an OD_600_ of 0.5. After 14 days of incubation, no significant differences in *C. vulgaris* G1-G cell count were measured between the control samples and co-cultures (Figure 2A). Co-cultivation of *S. vacuolatus* G1-O and all tested methylobacteria led to a significant increase in algal cell count after 7 days of incubation, independent from the initial bacterial cell load; however, a higher initial cell density of both *M. goesingense* strains resulted in even better performance of the microalga after 7 days of co-culturing. After 14 days of incubation, the effect of algal-growth promotion diminished; significant increase in algal cell count was only observable for co-cultures with *M. goesingense* Vab1 with an initial bacterial OD_600_ of 0.5 and for co-cultures with *M. goesingense* Vab2 (Figure 2B). The effect of microalgae growth-promotion was greatest for *H. lacustris;* co-culturing with an initial bacterial optical density of 0.5 led to a 4-fold, 12-fold, 14-fold, and 12-fold amount of biomass for *M. extorquens* Rab1, *M. mesophilicum* Sab1, *M. goesingense* Vab1 and *M. goesingense* Vab2 respectively when compared to the control group. Co-inoculation with a lower load of bacterial cells (OD_600_ 0.2) led only in the case of *M. mesophilicum* Sab1 to a significant increase in *H. lacustris* biomass (3.5-fold) after 7 days of incubation (Figure 2C). The growth rate of *H. lacustris* increased by 51% when co-cultivated with *M. goesingense* Vab1; this was the highest observed increase for all tested combinations. Algal CFUs for each treatment are provided in detail in Supplementary Tables S2A, S3A, and S4A together with the corresponding fluorescence intensities in Supplementary Tables S2B, S3B, and S4B.

### Visualization of Mixed Cultures Using Confocal Laser Scanning Microscopy

**FIGURE 3:**
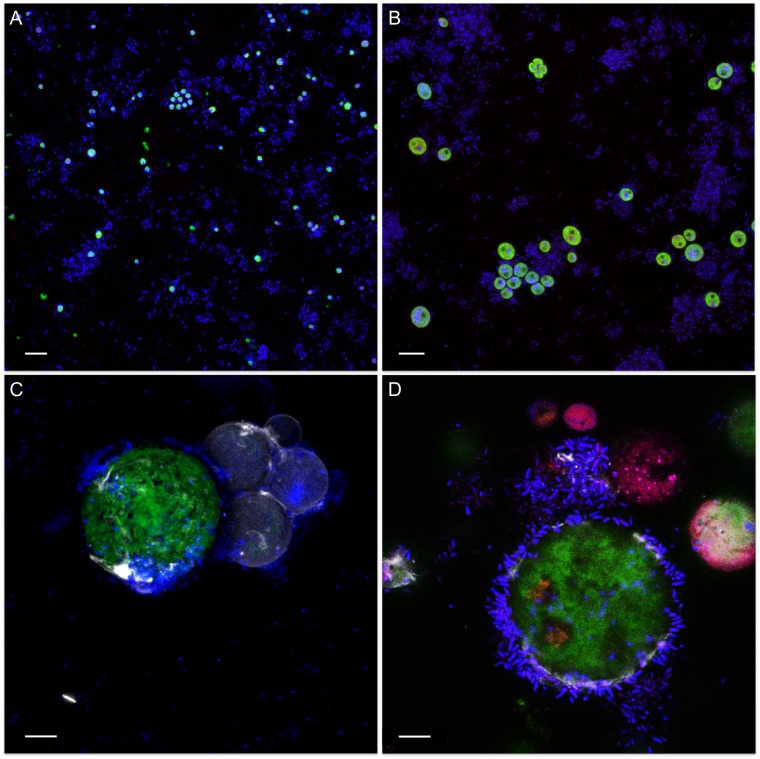
Confocal laser scanning micrographs of algae-bacteria cultures. The micrographs show different algae (**A:** *C. vulgaris* G1-G; **B:** *S. vacuolatus* G1-O; **C,D**: *H. lacustris* G1-R) that were inoculated with *M. goesingense* Vab1 with an initial optical density of 0.5 and visualized after 7 days of incubation. The microbial communities were stained with the LIVE/DEAD bacterial viability kit (Thermo Fisher Scientific, MA, United States) that allows differentiating between living and dead cells in combination with calcofluor white (0.15%) to enhance fluorescence of the algae. Green: autofluorescence of algae; blue: living cells; red: dead cells. Scale bar: 10 μm.

Visualization of mixed cultures revealed very loose associations between methylobacteria and *C. vulgaris* G1-G. Here, methylobacteria and algae were randomly and evenly distributed in the media and no distinct clustering was observed (Figure 3A). On the contrary, methylobacteria seem to embed *S. vacuolatus* cells and form cell aggregates (Figure 3B) and were found closely attached to the surface of viable *H. lacustris* cells (Figures 3C,D). Methylobacteria were also found in close proximity to disrupted, leaking *H. pluvialis* cells, seemingly feeding on microalgal cell debris (Figure 3D).

### Whole Genome Sequencing of Methylobacteria Provides Indication for Various Beneficial Traits

**FIGURE 4:**
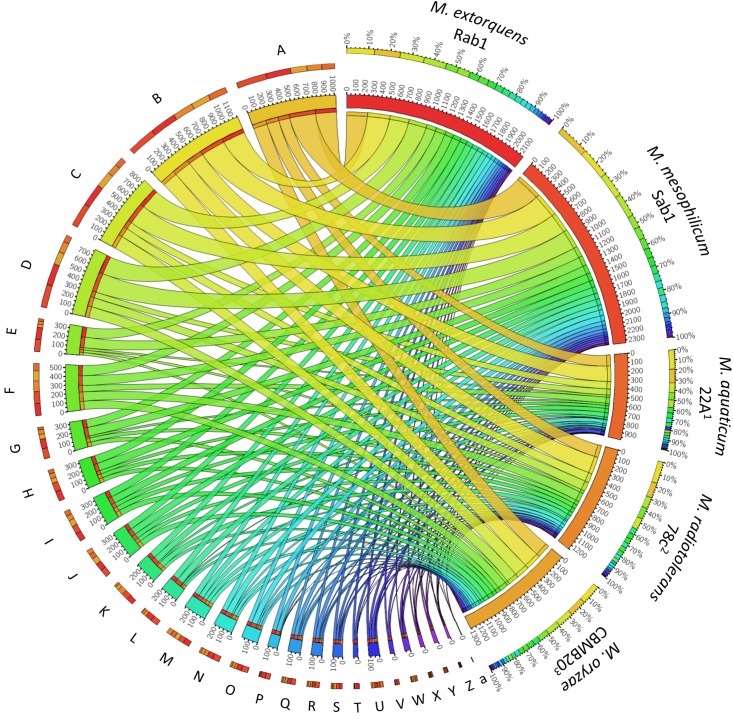
Feature distribution within the analyzed *Methylobacterium* genomes and reference genomes of plant-associated methylobacteria. The two algae-growth-promoting isolates *M. extorquens* Rab1 and *M. mesophilicum* Sab1 were compared with other isolates in order to identify distinct genetic features. Detected genes were grouped according to hierarchical assignment to SEED functions and visualized with Circos (http://circos.ca) to highlight the distribution of functional categories in each genome. A detailed feature count per organism per subcategory is included in Table 2 according to the SEED database after annotation using RAST (Aziz et al., 2008). Strain references: ^1^Kwak et al., 2014, ^2^Tani et al., 2015, ^3^Eevers et al., 2015.

The total genome sizes were determined to be 5,747,629 bp, featuring a GC content of 68.2% (*M. extorquens* Rab1) and 6,367,318 bp, with a GC content of 68.6% (*M. mesophilicum* Sab1). The genome analyses revealed 5,952 and 7,795 coding sequences for *M. extorquens* Rab1 and *M. mesophilicum* Sab1, respectively. Detailed statistics are provided in Table 1 including reference genomes of plant-associated methylobacteria. Identified features were assigned to subcategories with the SEED database (Overbeek et al., 2005). The analyzed genomes harbored between 221 (*M. extorquens* Rab1) and 234 (*M. mesophilicum* Sab1) genes associated with the synthesis of cofactors, vitamins, prosthetic groups and pigments, including genes responsible for the synthesis of biotin, riboflavin, tetrapyrroles as well as folate. Between 18 and 23 genes were related to iron acquisition and metabolism, whereas *M. extorquens* Rab1 harbored eleven and *M. mesophilicum* Sab1 16 genes associated with the production of siderophores. Both investigated methylobacteria harbored four genes associated with the synthesis of auxins. Details related to the distribution of features within both analyzed genomes as well as reference genomes of other plant-associated methylobacteria are displayed in Figure 4 and Table 2, including detailed feature counts per organism per subcategory.

**TABLE 1:** Detailed genome statistics for the analyzed *Methylobacterium* isolates.

	*M. extorquens* Rab1	*M. mesophilicum* Sab1	*M. oryzae* CBMB20^1^	*M. aquaticum* 22A^2^	*M. radiotolerans* 78c^3^
Total genome size [bp]	5,747,629	6,367,318	6,286,629	5,348,274	6,788,652
GC content [%]	68.2	68.6	69.8	71.1	71.2
N50	252,941	132,863	−	−	57,566
L50	8	13	1	1	38
Number of scaffolds	343	1660	1	1	271
Number of features	6,004	7,852	6,308	5,315	7,790
Number of coding sequences	5,952	7,795	6,243	5,212	7,740
Number of RNAs	52	57	65	103	50

*The genomes were sequenced using Illumina NextSeq paired-end sequencing. Information about reference genomes of plant-associated methylobacteria is included. Strain references:^1^Kwak et al., 2014, ^2^Tani et al., 2015, ^3^Eevers et al., 2015.*

**TABLE 2:** Detailed feature count for each microorganism per subcategory according to the SEED database after annotation using RAST (Aziz et al., 2008).

	Subcategory	*M. extorquens* Rab1	*M. mesophilicum* Sab1	*M. aquaticum* 22A^1^	*M. radiotolerans* 78c^2^	*M. oryzae* CBMB20^3^
A	Carbohydrates	253	314	133	150	169
B	Amino Acids and Derivatives	272	294	189	210	192
C	Protein Metabolism	235	242	91	111	161
D	Cofactors, Vitamins, Prosthetic Groups, Pigments	221	234	73	113	131
E	RNA Metabolism	120	128	26	32	32
F	Respiration	124	119	76	111	103
G	Stress Response	98	105	25	47	51
H	Fatty Acids, Lipids and Isoprenoids	99	93	39	49	49
I	Nucleosides and Nucleotides	85	83	50	55	65
J	DNA Metabolism	78	82	53	46	66
K	Membrane Transport	74	70	24	60	36
L	Cell Wall and Capsule	71	77	8	23	29
M	Motility and Chemotaxis	63	60	47	47	47
N	Virulence, Disease and Defense	59	71	21	42	29
O	Regulation and Cell signaling	49	68	22	27	24
P	Sulfur Metabolism	48	49	10	3	4
Q	Miscellaneous	35	46	16	27	18
R	Phosphorus Metabolism	31	43	13	20	19
S	Nitrogen Metabolism	30	28	12	16	27
T	Cell Division and Cell Cycle	25	24	2	2	2
U	Metabolism of Aromatic Compounds	25	27	34	16	20
V	Iron acquisition and metabolism	18	23	0	5	0
W	Phages, Prophages, Transposable elements Plasmids	7	21	6	17	15
X	Potassium metabolism	11	12	5	7	7
Y	Photosynthesis	11	11	0	10	10
Z	Secondary Metabolism	5	6	5	5	6
a	Dormancy and Sporulation	1	3	0	1	1

*Strain references: ^1^Kwak et al., 2014, ^2^Tani et al., 2015, ^3^Eevers et al., 2015.*

## Discussion

Our study revealed a co-occurrence of microalgae and methylobacteria in a natural biofilm when the whole bacterial community was assessed with high-throughput sequencing. Although the identified bacterial features assigned to the genus *Methylobacteria* occurred in rather low abundances, when compared to other bacterial constituents, we hypothesized that a synergistic relationship might occur between microalgae and distinct members of this genus. This is mainly due to their known association with land plants and the K-strategic lifestyle of methylobacteria based on their capability to grow on C1-compounds as sole carbon and energy source possibly exuded by algae. The synergistic relationship was confirmed by implementing different model organisms in co-cultivation experiments. Plant-associated methylobacteria were shown to significantly increase biomass formation at laboratory scale of two industrially relevant green algae in co-cultivation experiments. Genome analyses of the employed *M. extorquens* and *M. mesophilicum* strains revealed a number of features attributable to the observed algae growth-promotion, including genes involved in the production of vitamins, siderophores and auxins. The findings are supported by previous studies, where methylobacteria were identified in the phycosphere of *Chlorella, Scenedesmus*, *Micrasterias*, and *Chlamydomonas* (Levy et al., 2009; Krohn-Molt et al., 2017; Calatrava et al., 2018).

When different combinations were assessed, our co-cultivation experiments provided evidence that methylobacteria can stimulate the growth of certain microalgae through a species-specific relationship. In addition, also negative effects were observed for distinct algae-bacteria combinations and co-incubation periods. After 7 days of incubation, a significant decrease in biomass formation was observed for *C. vulgaris* irrespective of the applied methylobacteria. We assume that *C. vulgaris* and methylobacteria might compete for nutrients during the initial growth phase, resulting in a growth inhibition of the algae. Another possible explanation for the lower algal biomass when co-cultured with methylobacteria might be increased shading which attenuates light penetration (Kazamia et al., 2012). Carotenoids are known to have not only photoprotective capacities, but are also involved in the light-harvesting process during photosynthesis by expanding the light absorption spectrum. Detailed analyses of the methylobacteria genomes revealed that both investigated strains harbored genes related to the synthesis of bacterial light-harvesting proteins, as well as the photosynthetic reaction center of the photosystem type-II. Therefore, the beta-carotenoid-containing methylobacteria might also hamper photosynthetic activity of *Chlorella* through the competition for light (Van Dien et al., 2004). Although methylobacteria are able to produce vitamin B_12_ – an essential compound for many algae – Croft and colleagues could show that many microalgal species belonging to the genus *Chlorella* do not necessarily require cobalamin for proliferation (Croft et al., 2006). Moreover, the obtained visualizations of co-cultivated microorganisms revealed very loose associations between algae and methylobacteria in the case of *C. vulgaris.* The fact, that methylobacteria are not accumulated in the phycosphere of *C. vulgaris*, and thus impede a direct metabolite exchange, might also hamper a successful symbiotic relationship. Taken all these considerations into account, we suppose that the negative effects due to competition for nutrients, light and space outweigh the possible beneficial impact of the bacteria through the production of vitamins, auxins and siderophores for this bacteria-microalgae combination.

More distinctive effects were observed for *S. vacuolatus*; a significant increase in biomass formation was observed when it was co-cultured with methylobacteria after 7 days of incubation. The highest increase in biomass formation was achieved with both *M. goesingense* strains when they were added with an initial bacterial optical density of 0.5. While after 14 days the growth promoting effect of *M. extorquens* and *M. mesophilicum* was lost, significantly more algal biomass formation was still monitored in co-cultures with both *M. goesingense* strains, underlining the specificity of bacteria-microalgae symbioses. *M. goesingense* might metabolize the available nutrients more efficiently or harbors features which allows it to reduce the dissolved organic carbon produced by *S. vacuolatus* and thus the growth promoting effect was observed for prolonged time periods. Confocal laser scanning microscopy revealed algae cells that appeared to be embedded in bacterial aggregates. Similar colonization patterns were previously observed with methylobacteria and *Chlamydomonas*, where bacteria enable the growth of algae in a nitrogen depleted medium by mineralizing certain amino acids and peptides and thereby produce ammonium which consequently can be assimilated by the algae (Calatrava et al., 2018).

The algae growth-promoting effect of all investigated methylobacteria was strongest for *H. lacustris*, where up to 14-fold more algal biomass was formed after 7 days of incubation compared to the control. As reported by Croft et al. (2006), many *Haematococcus* species are dependent on an external supply of vitamins. In a vitamin-depleted medium, those essential micro-nutrients are provided by methylobacteria, allowing the microalgae to thrive. Visualization of *H. lacustris* co-cultures revealed that symbiotic bacteria appeared in close proximity of the microalgae, seemingly attached to the algal surface, allowing direct metabolite exchange. Similar colonization patterns of methylobacteria were observed on higher land plants, where bacteria are found attached to surface areas on leafs, attracted by the emitted methanol toward the stomata. The methanol produced by plants is a result of pectin-methylesterases de-esterifying the pectin within the cell wall during growth (Fall and Benson, 1996; Kutschera, 2007). During its life cycle, *H. lacustris* undergoes different cell stages; vegetative, motile cells become spherical, non-motile cyst cells under unfavorable conditions (Wayama et al., 2013). After 7 days of incubation, only a few flagellated cells were observable, while mostly green coccoid cells (palmelloid) and transitioning cells were found. In these stages, the microalgae change their extracellular matrix during the formation of a primary cell wall, which results in positive calcofluor-white staining indicating the presence of β-1,4-glycosidic linkages (Hagen et al., 2002). This was also evident during microscopic observations of the present study. Since the cell wall composition of *Haematococcus* is known to share similarities to those of plants, as they form primary and secondary cell walls consisting of cellulose and pectin along with other polysaccharides, a similar mechanism might be responsible for the attraction of methylobacteria in their surroundings (Wang et al., 2004). We found the highest growth-promoting effects for *H. lacustris* indicating that physical proximity is important for successful metabolite exchange and thus growth enhancement, as already suggested in previous studies (Gonzalez and Bashan, 2000; Hernandez et al., 2009). It is noteworthy to mention that the co-cultivation of microalgae and bacteria was not conducted under optimal conditions for any of the involved microorganisms. Nevertheless, this reflects conditions that prevail during large-scale cultivations where optimal light exposure and aeration are economically limited.

Microscopic observation of the analyzed biofilm revealed *H. lacustris* as the predominant algal species, which has likely an effect on the prevalent bacterial community. Natural co-occurrences of *H. lacustris* and methylobacteria, as revealed through 16S rRNA gene fragment amplicon sequencing, support the hypothesis of specific, evolutionary evolved co-occurrences of certain microalgae and bacteria in natural habitats. Complementary analysis of the two methylobacteria genomes revealed that both strains harbor several features which have already been described to positively contribute to algae growth including genes involved in the production of a variety of vitamins, such as cobalamin, biotin, thiamin and riboflavin, indicating the potential of methylobacteria to support algae growth. Reference data from other strains revealed that those genes are frequently found in different *Methylobacterium* spp., underlining the potential of methylobacteria as promising growth promoters of algae. In addition to genes involved in the synthesis of vitamins, genes attributable to the production of plant hormones were found in the genomes of the utilized model strains. Several studies have demonstrated that phytohormones can stimulate the growth and lipid production of microalgae (de-Bashan et al., 2008; Amin et al., 2015; Liu et al., 2016; Yu et al., 2017). Additionally, both genomes contained a substantial higher number of genes involved in iron acquisition and metabolism than genomes of other plant-associated methylobacteria that were implemented as a reference. It was previously shown that microalgae benefit from bacterial siderophores as the bioavailability of chelated iron is increased resulting in a “carbon for iron mutualism” where algae assimilate iron complexed in bacterial siderophores and in return provide the for the bacteria essential dissolved organic matter (Amin et al., 2009, 2012, 2015).

Our results provide new insights into the potential of plant-associated methylobacteria to promote the growth of two different, industrially relevant microalgal genera. Moreover, the observations lead us to the conclusion that symbiotic relationships are interchangeable between plant and microalgae hosts, but selective in terms of species specificity. In addition, our results provide evidence that a balanced ratio between symbiotic bacteria and microalgae is essential for algae growth-promotion, which facilitates a mutualistic relationship without interferences through the competition for nutrients, light and space. Deepening exploration of natural algae-bacteria relationships will further facilitate the design of synthetic microbial communities, which are a promising tool for biotechnology and might lead to improved cultivation procedures in the future.

## Data Availability Statement

The amplicon datasets used and/or analyzed during the current study are available in the ENA repository (https://www.ebi.ac.uk/ena) under the accession number PRJEB35130; both genome assemblies are available in the ENA repository under the accession number PRJEB35220; and the sequences of the implemented algae strains were deposited in the ENA repository under accession number PRJEB36220.

## Author Contributions

GB developed the study design. LK conducted the environmental sampling, performed the laboratory experiments and the bioinformatics analyses. HM processed the genomes. CD and CM discussed results. GB, LK, and TC interpreted the data and wrote the manuscript. All authors reviewed and approved the final version of the manuscript.

## Conflict of Interest

LK was employed by the company acib GmbH. CM and CD were employed by company bio-ferm GmbH. The remaining authors declare that the research was conducted in the absence of any commercial or financial relationships that could be construed as a potential conflict of interest.
