# Supplementary material for: Plant Growth-Promoting Methylobacteria Selectively Increase the Biomass of Biotechnologically Relevant Microalgae
Source: Front Microbiol. 2020 Mar 18;11:427. doi: 10.3389/fmicb.2020.00427 (PMC7093331; doi:10.3389/fmicb.2020.00427)
Supplement: Supplementary file 1 [file Data_Sheet_1.docx]

***Supplementary Material***

**Plant growth-promoting methylobacteria increase the biomass of biotechnologically relevant microalgae**

**Lisa Krug**^1^**^,2^, Christina Morauf^3^, Christina Donat^3^, Henry Müller^1^, Tomislav Cernava^1*^ and Gabriele Berg^1^**

^1^Institute of Environmental Biotechnology, Graz University of Technology, Petersgasse 12, 8010 Graz, Austria

^2^ACIB GmbH, Petersgasse 14, 8010 Graz, Austria

^3^bio-ferm GmbH, Erber Campus 1, 3131 Getzersdorf, Austria

**
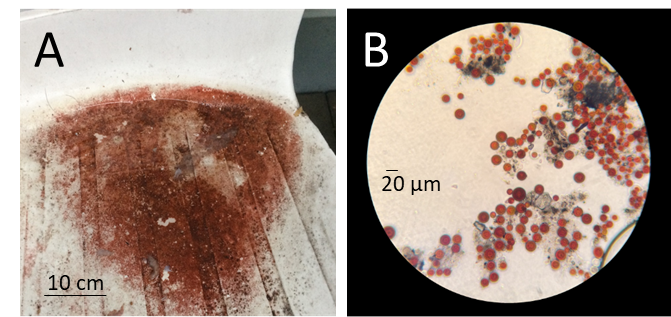
**

**Supplementary Figure S1.** **Macroscopic and microscopic visualization of the sampled biofilm with a typical coloration for microalgae-dominated communities.** Samples obtained from outdoor furniture showed indications for the presence of *Haematococcus* sp. due to the specific red color (A). A microscopic observation confirmed a high abundance of microalgae in the obtained samples (B).


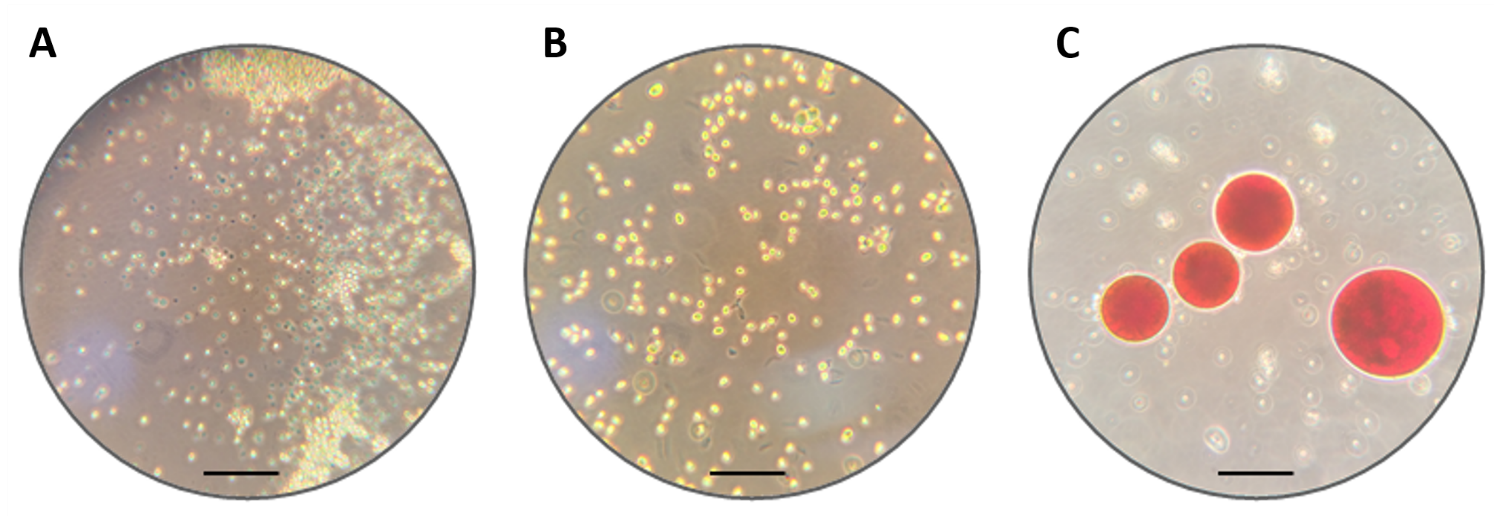


**Supplementary Figure S2.** **Micrographs of isolated algae from the biofilm.** *C. vulgaris* G1-G (A), *S. vacuolatus* G1-O (B) and *H. lacustris* G1-R (C) were isolated from a microalgae dominated biofilm. Pure cultures were obtained by subcultivation. Scale bar: 20 µm


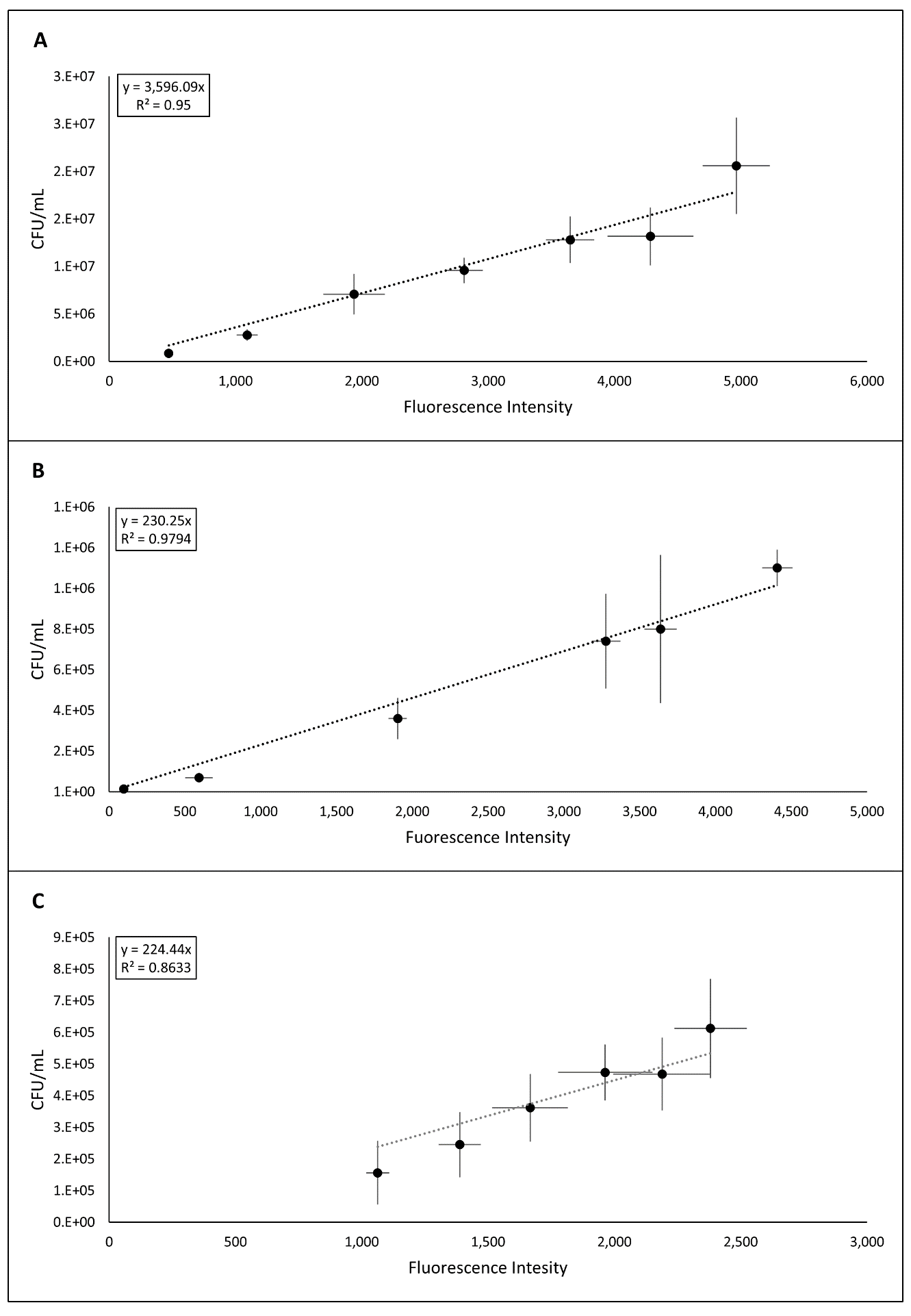


**Supplementary Figure S3.** **Indirect determination of algal cell counts with fluorescence intensity measurements.** Correlation between fluorescence intensity and algal cell count for *C. vulgaris* G1-G (A), *S. vacuolatus* G1-O (B) and *H. lacustris* G1-R (C).

**Supplementary Table S1. Closest alignment hits of features representing the genus *Methylobacterium***. Sequences were aligned against the NCBI nucleotide collection using the BLAST algorithm.

| **Feature ID** | **Closest NCBI hit** | **Accessions NO.** |
| --- | --- | --- |
| 3372941629954ad743c804e88345969d | *Methylobacterium indicum* strain DP28.3 | MK968432.1 |
| 6e98ad9b6beaa6c0546f6cc9b274d0fa | Uncultured *Methylobacterium* sp. | LC466937.1 |
| ac829040ac43f4d961a191fd8e5a915d | *Methylobacterium* sp. strain I-S-R3-1 | MK398052.1 |
| d8456fac09d0bb7dfd62de648ca0a933 | *Methylobacterium* sp. 14-324 | EF558714.1 |
| f58bba003c01c620836eafcae31a7198 | *Methylobacterium* sp. CSCXZN6.6 | LC484783.1 |

**Supplementary Table S2A.** Calculated *C. vulgaris* G1-G cell count after seven days and 14 days of incubation. Algal cell count of mixed cultures with differing initial bacterial cell densities (OD_600_ = 0.2 and 0.5) were compared with control cultures where no additional bacteria were added. Significances were calculated using ANOVA for normally distributed values and the Kruskal-Wallis test for non-parametric analyses including Bonferroni multiple test correction. Asterisk indicates significant differences (*p*-value ≤ 0.05) in algal cell count compared to control samples after the respective time of incubation.

|  | | | | | |  |
| --- | --- | --- | --- | --- | --- | --- |
| ***M. extorquens* Rab1** | | | | | |  |
|  | T7 [× 10^6^ CFU/mL] | difference in CFU count after 7 days | T14 [× 10^6^ CFU/mL] | difference in CFU count after 14 days |  |  |
| control | 2.16 ± 0.12 |  | 2.96 ± 0.54 |  |  |  |
| OD_600_ 0.2 | 1.74 ± 0.18* | -20% | 3.43 ± 0.37 | +16% |  |  |
| OD_600_ 0.5 | 1.78 ± 0.18* | -18% | 3.13 ± 0.42 | +6% |  |  |
|  |  |  |  |  |  |  |
| ***M. mesophilicum* Sab1** | | | | | |  |
|  | T7 [× 10^6^ CFU/mL] | difference in CFU count after 7 days | T14 [× 10^6^ CFU/mL] | difference in CFU count after 14 days |  |  |
| control | 2.16 ± 0.12 |  | 2.96 ± 0.54 |  |  |  |
| OD_600_ 0.2 | 1.81 ± 0.15* | -16% | 3.63 ± 0.66 | +23% |  |  |
| OD_600_ 0.5 | 1.90 ± 0.07 | -12% | 3.36 ± 0.30 | +14% |  |  |
|  |  |  |  |  |  | |
| ***M. goesingense* Vab1** | | | | | |  |
|  | T7 [× 10^6^ CFU/mL] | difference in CFU count after 7 days | T14 [× 10^6^ CFU/mL] | difference in CFU count after 14 days |  |  |
| control | 2.16 ± 0.12 |  | 2.96 ± 0.54 |  |  |  |
| OD_600_ 0.2 | 1.73 ± 0.22* | -20% | 2.98 ± 0.43 | +1% |  |  |
| OD_600_ 0.5 | 1.64 ± 0.21* | -24% | 3.23 ± 0.51 | +9% |  |  |
|  |  |  |  |  |  | |
| ***M. goesingense* Vab2** | | | | | |  |
|  | T7 [× 10^6^ CFU/mL] | difference in CFU count after 7 days | T14 [× 10^6^ CFU/mL] | difference in CFU count after 14 days |  |  |
| control | 2.16 ± 0.12 |  | 2.96 ± 0.54 |  |  |  |
| OD_600_ 0.2 | 1.88 ± 0.19* | -13% | 2.59 ± 0.56* | -12% |  |  |
| OD_600_ 0.5 | 2.08 ± 0.21 | -4% | 3.05 ± 0.43 | +3% |  |  |

**Supplementary Table S2B.** Fluorescence intensities obtained with *C. vulgaris* G1-G and four methylobacteria after seven days and 14 days of incubation.

| ***M. extorquens* Rab1** | | | |  |  |  |  |  |  |
| --- | --- | --- | --- | --- | --- | --- | --- | --- | --- |
|  | T7 | T14 | | |  |  |  |  |  |
| control | 601 ± 47 | 822 ± 150 | | |  |  |  |  |  |
| OD_600_ 0.2 | 483 ± 51 | 954 ± 103 | | |  |  |  |  |  |
| OD_600_ 0.5 | 496 ± 50 | 871 ± 116 | | |  |  |  |  |  |
|  |  |  | | |  |  |  |  |  |
| ***M. mesophilicum* Sab1** | | | | | |  |  |  |  |
|  | T7 | T14 | | |  |  |  |  |  |
| control | 601 ± 47 | 822 ± 150 | | |  |  |  |  |  |
| OD_600_ 0.2 | 504 ± 42 | 1009 ± 182 | | |  |  |  |  |  |
| OD_600_ 0.5 | 530 ± 21 | 934 ± 83 | | |  |  |  |  |  |
|  |  |  | | |  | |  | |  |
| ***M. goesingense* Vab1** | | | | | |  |  |  |  |
|  | T7 | T14 | | |  |  |  |  |  |
| control | 601 ± 47 | 822 ± 150 | | |  |  |  |  |  |
| OD_600_ 0.2 | 481 ± 61 | 829 ± 120 | | |  |  |  |  |  |
| OD_600_ 0.5 | 457 ± 60 | 899 ± 142 | | |  |  |  |  |  |
|  |  |  |  | | | |  |  | |
| ***M. goesingense* Vab2** | | | | | |  |  |  |  |
|  | T7 | T14 | | |  |  |  |  |  |
| control | 601 ± 47 | 822 ± 150 | | |  |  |  |  |  |
| OD_600_ 0.2 | 523 ± 52 | 720 ± 156 | | |  |  |  |  |  |
| OD_600_ 0.5 | 578 ± 57 | 921 ± 197 | | |  |  |  |  |  |

**Supplementary Table S3A.** Calculated *S. vacuolatus* G1-O cell count after seven days and 14 days of incubation. Algal cell count of mixed cultures with differing initial bacterial cell densities (OD_600_ = 0.2 and 0.5) were compared with control cultures where no additional bacteria were added. Significances were calculated using ANOVA for normally distributed values and the Kruskal-Wallis test for non-parametric analyses including Bonferroni multiple test correction. Asterisk indicates significant differences (*p*-value ≤ 0.05) in algal cell count compared to control samples after the respective time of incubation.

|  | | | | | |  |
| --- | --- | --- | --- | --- | --- | --- |
| ***M. extorquens* Rab1** | | | | | |  |
|  | T7  [× 10^5^ CFU/mL] | difference in CFU count after 7 days | T14 [× 10^5^ CFU/mL] | difference in CFU count after 14 days |  |  |
| control | 1.01 ± 0.01 |  | 2.56 ± 0.27 |  |  |  |
| OD_600_ 0.2 | 1.38 ± 0.12* | +36% | 3.04 ± 0.38 | +15% |  |  |
| OD_600_ 0.5 | 1.48 ± 0.14* | +47% | 2.93 ± 0.36 | +10% |  |  |
|  |  |  |  |  |  |  |
| ***M. mesophilicum* Sab1** | | | | | |  |
|  | T7 [× 10^5^ CFU/mL] | difference in CFU count after 7 days | T14 [× 10^5^ CFU/mL] | difference in CFU count after 14 days |  |  |
| control | 1.01 ± 0.01 |  | 2.56 ± 0.27 |  |  |  |
| OD_600_ 0.2 | 1.31 ± 0.11* | +30% | 2.71 ± 0.27 | +2% |  |  |
| OD_600_ 0.5 | 1.30 ± 0.12* | +28% | 2.85 ± 0.39 | +8% |  |  |
|  |  |  |  |  |  | |
| ***M. goesingense* Vab1** | | | | | |  |
|  | T7 [× 10^5^ CFU/mL] | difference in CFU count after 7 days | T14 [× 10^5^ CFU/mL] | difference in CFU count after 14 days |  |  |
| control | 1.01 ± 0.01 |  | 2.56 ± 0.27 |  |  |  |
| OD_600_ 0.2 | 1.35 ± 0.14* | +34% | 3.02 ± 0.56 | +14% |  |  |
| OD_600_ 0.5 | 1.73 ± 0.23* | +72% | 3.49 ± 0.36* | +32% |  |  |
|  |  |  |  |  |  | |
| ***M. goesingense* Vab2** | | | | | |  |
|  | T7 [× 10^5^ CFU/mL] | difference in CFU count after 7 days | T14 [× 10^5^ CFU/mL] | difference in CFU count after 14 days |  |  |
| control | 1.01 ± 0.01 |  | 2.56 ± 0.27 |  |  |  |
| OD_600_ 0.2 | 1.29 ± 0.13* | +28% | 3.36 ± 0.53* | +27% |  |  |
| OD_600_ 0.5 | 1.62 ± 0.25* | +60% | 3.48 ± 0.68* | +32% |  |  |

**Supplementary Table S3B.** Fluorescence intensities obtained with *S. vacuolatus* G1-O and four methylobacteria after seven days and 14 days of incubation.

| ***M. extorquens* Rab1** | | | |  |  |  |  |  |  |
| --- | --- | --- | --- | --- | --- | --- | --- | --- | --- |
|  | T7 | T14 | | |  |  |  |  |  |
| control | 437 ± 42 | 1150 ± 119 | | |  |  |  |  |  |
| OD_600_ 0.2 | 597 ± 52 | 1319 ± 165 | | |  |  |  |  |  |
| OD_600_ 0.5 | 644 ± 62 | 1272 ± 156 | | |  |  |  |  |  |
|  |  |  | | |  |  |  |  |  |
| ***M. mesophilicum* Sab1** | | | | | |  |  |  |  |
|  | T7 | T14 | | |  |  |  |  |  |
| control | 437 ± 42 | 1150 ± 119 | | |  |  |  |  |  |
| OD_600_ 0.2 | 570 ± 49 | 1178 ± 117 | | |  |  |  |  |  |
| OD_600_ 0.5 | 563 ± 54 | 1239 ± 171 | | |  |  |  |  |  |
|  |  |  | | |  | |  | |  |
| ***M. goesingense* Vab1** | | | | | |  |  |  |  |
|  | T7 | T14 | | |  |  |  |  |  |
| control | 437 ± 42 | 1150 ± 119 | | |  |  |  |  |  |
| OD_600_ 0.2 | 588 ± 61 | 1435 ± 298 | | |  |  |  |  |  |
| OD_600_ 0.5 | 753 ± 98 | 1515 ± 155 | | |  |  |  |  |  |
|  |  |  |  | | | |  |  | |
| ***M. goesingense* Vab2** | | | | | |  |  |  |  |
|  | T7 | T14 | | |  |  |  |  |  |
| control | 437 ± 42 | 1150 ± 119 | | |  |  |  |  |  |
| OD_600_ 0.2 | 561 ± 56 | 1460 ± 231 | | |  |  |  |  |  |
| OD_600_ 0.5 | 702 ± 110 | 1514 ± 296 | | |  |  |  |  |  |

**Supplementary Table S4A.** Calculated *H. lacustris* G1-R cell count after seven days of incubation. Algal cell count of mixed cultures with differing initial bacterial cell densities (OD_600_ = 0.2 and 0.5) were compared with control cultures where no additional bacteria were added. Significances were calculated using ANOVA for normally distributed values and the Kruskal-Wallis test for non-parametric analyses including Bonferroni multiple test correction. Asterisk indicates significant differences (*p*-value ≤ 0.05) in algal cell count compared to control samples after the respective time of incubation.

|  | | | | | |
| --- | --- | --- | --- | --- | --- |
| ***M. extorquens* Rab1** | | | | | |
|  | T7  [× 10^4^ CFU/mL] | difference in CFU count after 7 days | |  |  |
| control | 2.00 ± 0.29 |  | |  |  |
| OD_600_ 0.2 | 2.44 ± 0.54 | +22% | |  |  |
| OD_600_ 0.5 | 8.15 ± 1.77* | +308% | |  |  |
|  |  |  |  | |  |
| ***M. mesophilicum* Sab1** | | | | | |
|  | T7 [× 10^4^ CFU/mL] | difference in CFU count after 7 days | |  |  |
| control | 2.00 ± 0.29 |  | |  |  |
| OD_600_ 0.2 | 7.06 ± 1.63* | +254% | |  |  |
| OD_600_ 0.5 | 24.03 ± 0.24* | +1,104% | |  |  |
|  |  |  | |  |  |
| ***M. goesingense* Vab1** | | | | | |
|  | T7 [× 10^4^ CFU/mL] | difference in CFU count after 7 days | |  |  |
| control | 2.00 ± 0.29 |  | |  |  |
| OD_600_ 0.2 | 3.68 ± 1.27 | +84% | |  |  |
| OD_600_ 0.5 | 28.08 ± 0.81* | +1,307% | |  |  |
|  |  |  | |  |  |
| ***M. goesingense* Vab2** | | | | | |
|  | T7  [× 10^4^ CFU/mL] | difference in CFU count after 7 days | |  |  |
| control | 2.00 ± 0.29 |  | |  |  |
| OD_600_ 0.2 | 3.22 ± 0.86 | +61% | |  |  |
| OD_600_ 0.5 | 24.14 ± 2.81* | +1,109% | |  |  |

**Supplementary Table S4B.** Fluorescence intensities obtained with *H. lacustris* G1-R and four methylobacteria after seven days of incubation.

| ***M. extorquens* Rab1** | |  |  |  |  |  |  |
| --- | --- | --- | --- | --- | --- | --- | --- |
|  | T7 | |  |  |  |  |  |
| control | 89 ± 13 | |  |  |  |  |  |
| OD_600_ 0.2 | 109 ± 24 | |  |  |  |  |  |
| OD_600_ 0.5 | 364 ± 79 | |  |  |  |  |  |
|  |  | |  | |  |  |  |
| ***M. mesophilicum* Sab1** | | | |  |  |  |  |
|  | T7 | |  |  |  |  |  |
| control | 89 ± 13 | |  |  |  |  |  |
| OD_600_ 0.2 | 315 ± 73 | |  |  |  |  |  |
| OD_600_ 0.5 | 1073 ± 106 | |  |  |  |  |  |
|  |  | |  | |  | | |
| ***M. goesingense* Vab1** | | | |  |  |  |  |
|  | T7 | |  |  |  |  |  |
| control | 89 ± 13 | |  |  |  |  |  |
| OD_600_ 0.2 | 164 ± 57 | |  |  |  |  |  |
| OD_600_ 0.5 | 1254 ± 215 | |  |  |  |  |  |
|  |  | |  | | |  |  |
| ***M. goesingense* Vab2** | | | |  |  |  |  |
|  | T7 | |  |  |  |  |  |
| control | 89 ± 13 | |  |  |  |  |  |
| OD_600_ 0.2 | 172 ± 101 | |  |  |  |  |  |
| OD_600_ 0.5 | 1078 ± 126 | |  |  |  |  |  |
